# Relationship between menstrual cycle and cognitive function in women with primary dysmenorrhea

**DOI:** 10.55730/1300-0144.5966

**Published:** 2024-12-05

**Authors:** Ufuk ATLIHAN, Meltem DURAKLI ULUKÖK, Onur YAVUZ

**Affiliations:** 1 Department of Gynecology and Obstetrics, Private Karataş Hospital, İzmir, Turkiye; 2 Department of Neurology, Private Cankaya Medical Center, İzmir, Turkiye; 3 Department of Gynecology and Obstetrics, Faculty of Medicine, Dokuz Eylul University, İzmir, Turkiye

**Keywords:** Cognitive function, menstrual cycle, primary dysmenorrhea

## Abstract

**Background/aim:**

To evaluate the relationship between the change in pain intensity over days of the menstrual cycle and cognitive function in females with primary dysmenorrhea.

**Materials and methods:**

One hundred thirty-four females with primary dysmenorrhea who were admitted to our hospital from January 2021 to November 2023 were assessed. Of these participants, 46 individuals who subjectively stated that their cognitive function was affected, for whom neurology consultation was requested, and for whom patient data were available, were included in the study.

**Results:**

A significant decrease was seen in phonemic and semantic fluency on day 3 of menstruation (p < 0.001 and p < 0.001, respectively). A significant decrease was observed in the forward and backward digit span (DGS) scores on day 3 of menstruation (p < 0.001 and p < 0.001, respectively). A significant decrease was seen in the forward and backward DGS scores on the most painful day of menstruation (p < 0.001 and p < 0.001, respectively). A significant decrease in the logical memory subtest Wechsler Memory Scale revised scores on the most painful day of menstruation (p < 0.001).

**Conclusion:**

Consistent with the literature, strong evidence was found showing a negative correlation between dysmenorrhea and cognitive function. Future prospective, larger sample-size studies comparing cognitive function in females with and without dysmenorrhea at various stages of the menstrual cycle are needed.

## 1. Introduction

The most prevalent gynecologic condition in women, dysmenorrhea, significantly impairs their quality of life [1]. It has a significant influence on women’s mental health and physical well-being and has serious implications for their personal relationships, educational life, and work performance. However, if dysmenorrhea is left untreated for a long time, hyperalgesia may develop and secondarily, a tendency toward chronic pelvic pain may occur [1]. Pain that occurs during menstruation without any accompanying disease is called primary dysmenorrhea. The pathophysiology of primary dysmenorrhea is probably due to the increased production of prostanoids, especially prostaglandins, by the cyclooxygenase pathway [2]. Secondary dysmenorrhea is usually seen in females aged 30–40 years, it has the same symptoms as painful menstruation, and there is always an underlying organic pathology [3–5]. Irregular menstrual cycles, nulliparity, early menstruation, prolonged menstruation, excessive menstrual bleeding, smoking, and a family record of dysmenorrhea are some of the characteristics that increase the risk of primary dysmenorrhea [6].

Primary dysmenorrhea generally has psychogenic consequences in addition to medical symptoms. Investigations in the literature have shown that women with dysmenorrhea generally have a lower quality of life than healthy women and that a strong relationship exists between the intensity of dysmenorrhea and their quality of life [7–9]. The frequency of primary dysmenorrhea in those of reproductive age varies between 45% and 95%, and 2%–29% of them describe severe pain during menstruation [10,11]. According to Burnett et al. [12], 51% experienced restrictions in different daily activities, 17% were unable to attend work or school because of discomfort, and 60% of those over the age of 18 with primary dysmenorrhea had significant or at least mild discomfort.

It has been demonstrated that primary dysmenorrhea significantly impairs everyday activities, academic performance, work productivity, and quality of life [13,14]. About 42% of young girls in the study of Alldredge et al. [14] stated that primary dysmenorrhea limited their ability to do everyday tasks, and 17% said they missed one or two days of work or school. According to their research, women with primary dysmenorrhea had a considerably greater frequency of depression and anxiety than those in good health, and a correlation was found between primary dysmenorrhea and the presence of stress [15,16]. Given the positive association between the overall pain experience, regardless of location, and episodic deterioration of cognitive function, it is likely that women who describe dysmenorrhea experience a transient decline in cognitive function while experiencing pain [17–19]. The literature contains research that demonstrates how dysmenorrhea impairs overall functioning [20,21]. These studies have shown that women’s attention deficit increases during menstrual periods, and they tend to make more mistakes. However, data about cognitive performance in women with primary dysmenorrhea are still unclear.

The majority of research on the cognitive effects of dysmenorrhea compares women with and without symptoms in cross-sectional studies. Fluctuations in cognitive function throughout the menstrual cycle have not been assessed. Munro et al. [22] revealed that female university students’ menstrual experiences might negatively affect their education. Prior research has concentrated on the impact on cognitive functions between the presence and absence of primary dysmenorrhea, but has not focused on the effects and fluctuations on cognitive function within the menstrual cycle process itself. Hence, it was herein aimed to evaluate the relationship between the change in pain intensity over the days of the menstrual cycle and cognitive function in females with primary dysmenorrhea.

## 2. Materials and methods

### 2.1. Study design and participants

In accordance with the principles of the Helsinki Declaration, the current investigation was planned as a retrospective observational study. All the patients provided signed informed consent forms. The study received approval from the Ethics Committee of our hospital (Date: 09/10/24, Number: 2024/33-04). In this current investigation, 134 females with primary dysmenorrhea who were admitted to our hospital from January 2021 to November 2023 were assessed. Of these participants, 46 who subjectively stated that their cognitive function was affected, for whom neurology consultation was requested, and for whom patient data were available, were included in the study. Participants who met the inclusion criteria were required to declare that for painful menstruation, they had at least one symptom from each of the two suggested symptom groups. The primary symptoms used in the evaluation were as follows: pain symptoms (abdominal pain, internal organ cramps in the lower abdomen, lower back pain, hip pain, and groin pain). Secondary symptoms included fainting or fatigue, diarrhea, vomiting and nausea, and concentration problems [13,23]. Patients diagnosed with neurologic and/or psychiatric diseases, those using medications that could affect their speed of thinking (antipsychotics, benzodiazepines, and barbiturate group drugs), those using alcohol and/or drugs, those with pelvic area diseases or any comorbidities, and those using any painkillers during menstrual periods were excluded from the study.

The pain scores of all the participants, as assessed using a visual analog scale (VAS) between days 2 and 5 of the menstrual period, were scanned separately for each day. All tests performed for cognitive function between days 2 and 5 of menstruation were evaluated retrospectively.

Verbal fluency tests were applied to participants with primary dysmenorrhea to evaluate executive function and complex attention. The test had two parts: semantic fluency and phonemic fluency [24]. The digit span (DGS) test was employed to make an evaluation regarding instant memory and complex attention function. It is one of the subtests in the revised form of the Wechsler Memory Scale revised (WMS-R) and consists of two main sections: forward and backward DGS [25]. For making an evaluation regarding verbal memory, the logical memory subtest of the WMS-R was applied [25]. Karakaş et al. [26] conducted the standardization study of WMS-R for Turkish culture.

### 2.2. Statistical analysis

IBM SPSS Statistics for Windows 26.0 (IBM Corp., Armonk, NY, USA) was used for statistical analysis. The Kolmogorov-Smirnov test was used to assess the normality of the distribution. The Mann Whitney-U and Kruskal–Wallis post hoc tests were used to assess parameters that were not normally distributed. Quantitative data were presented as the mean ± standard deviation (SD) (minimum–maximum) or median (minimum–maximum). Spearman correlation analysis (r) was performed to determine correlations between variables. A 95% confidence interval (CI) was used to analyze the results. Statistical significance was accepted as p < 0.05.

## 3. Results

The average age of the participants was 26.1 ± 8.4 years and the average body mass index was 24.3 ± 3.2 kg/m^2^. As seen in Table 1, there was a significant decrease in phonemic and semantic fluency in on day 3 of menstruation (p < 0.001 and p < 0.001, respectively). A significant decrease was seen in the forward and backward DGS scores on day 3 of menstruation (p < 0.001 and p < 0.001, respectively). A significant decrease was observed in the logical memory subtest of the WMS-R on day 3 of menstruation (p < 0.001). A significant increase was seen in the VAS (dysmenorrhea) scores on day 3 of menstruation (p < 0.001).

As seen in Table 2, a significant decrease was observed in the phonemic and semantic fluency on the most painful day of menstruation (p < 0.001 and p < 0.001, respectively). A significant decrease was seen in the forward and backward DGS scores on the most painful day of menstruation (p < 0.001 and p < 0.001, respectively). A significant decrease was seen in the logical memory subtest of the WMS-R on the most painful day of menstruation (p < 0.001).

As seen in Table 3, a significant negative correlation was seen between phonemic fluency and the VAS day 2 scores (p < 0.05, r = −0.9). A significant negative correlation was seen between semantic fluency and the VAS day 2 scores (p < 0.05, r = −0.8).

As seen in Table 4, a significant negative correlation was seen between the forward DGS and VAS day 2 scores (p < 0.05, r = −0.8). A moderate negative correlation was found between the backward DGS and VAS day 2 scores (p < 0.05, r = −0.5).

As seen in Table 5, a moderate negative correlation was found between the logical memory subtest of the WMS-R and VAS day 2 scores (p < 0.05, r = −0.5).

## 4. Discussion

Changes in cognitive functions experienced in women during their menstrual period depending on the presence and severity of dysmenorrhea was investigated herein. The menstrual period was evaluated by both the day and the most and least painful days of menstruation. In the evaluation, the relationship between cognitive function and the VAS (dysmenorrhea) scores was examined. The findings confirmed a connection between the VAS and cognitive function scores, revealing a negative correlation, while at the same time revealing the worst cognitive function on the most painful day. The results revealed an impairment in cognitive and inhibitory control; processing speed, processing, and task-switching speed; as well as reaction time, in addition to episodic verbal memory.

Keogh et al. [21] found that throughout menstruation, attentional interference effects were more evident. However, they reported that the basis of this impact was a general deterioration in performance rather than a specific attention deficit. There has long been research on how the menstrual cycle affects cognitive performance and emotional state. This can be easily confirmed by the observation that menstruation is related to its emotional impact, especially during the premenstrual period [27]. However, there are also studies in the literature showing no difference in cognitive function or its subcomponents during the menstruation. Two mechanisms have been suggested for this. It may be that the skills tested were often basic or that these skills had become automatic and were therefore unaffected by pain. Another view is that the slower speed was due to a general slowing down of processing and a shorter period of focus [18,28]. Maki et al. [29] revealed that women scored lower on memory tasks during menstruation. Studies have also shown that longer reaction times and more verification before responding are caused by pain, which increases the attentional processing burden.

During the pain period, there was a higher strain on cognitive function, which had a negative effect on proper functioning [30,31]. The menstrual period is a physiologic process with various biopsychosocial points. There is a reflection of this period for women of all cultures and socioeconomic levels. During this stage, many women experience at least some mental and physical incoordination. Severe pain can cause stress, depressive mood, anxiety, and nervousness [15]. Barnard et al. [32] suggested that reduced cognitive performance during particular menstrual cycle stages may account for at least some of the association between pain sensation and worse psychosocial functioning in primary dysmenorrhea. According to Magni et al. [33], individuals with chronic pain were three times more likely than those without it to meet the criteria for depression. However, there are studies on the association between cognitive impairment and depression [34]. Long-term physical discomfort and suffering have a detrimental effect on cognitive function. It has been demonstrated that such experiences challenge the daily functions of women with dysmenorrhea and may be exacerbated by the effects of stress, negative emotional experiences, and depression-anxiety symptoms [35]. In a study by Maki et al. [29], women scored higher on memory tasks and those that required fine motor skills in the middle of the luteal phase and less throughout the menstrual phase. Lord et al. [36] evaluated the premenstrual period in their study and revealed low premenstrual scores in the concentration assessment. Hampson et al. [37] found that assessment scores on speed and motor coordination tests increased in the midluteal phase in contrast to the early follicular phase.

Many studies have evaluated cognitive function during the menstrual period, and there is strong evidence of the negative correlation between dysmenorrhea and cognitive function. However, the current study important information to the literature in terms of using many different function tests and evaluating each day of the menstrual period separately. Nevertheless, there were some limitations to the study, such as its retrospective design and small sample size. The possible impact of the hormone cycle on cognitive performance was another drawback that might complicate the connection between pain and cognitive function. It is crucial to keep in mind that hormonal processes may influence how pain is perceived, even if it does not diminish the significance of pain. The strength of the study was that the inclusion criterion for primary dysmenorrhea was determined after investigating all secondary dysmenorrhea etiologies. Primary dysmenorrhea was diagnosed clinically in all the patients. The majority of the participants in the study were aged 18–34 years; thus, the associations found have to be regarded as traits of individuals in the aforementioned age range. Considering the reproductive period, although it does not fully cover all patient groups in the age range, it provides a great idea of it. Patients with chronic pain experience increased self-reported pain due to negative mood, indicating the role of chronic pain in pain perception.

To determine the respective contributions of pain and mood on cognitive performance, future studies should employ questionnaires that measure both at the same time and enable a combined regression analysis. Our comprehension of the physical-mental interplay that the biopsychosocial model proposes might be improved by such an approach. Future prospective, larger sample-size studies comparing cognitive function in individuals with and without dysmenorrhea at various stages of the menstrual cycle are needed.

## Figures and Tables

**Table 1:** Comparison of the cognitive function scores across the days.

Variables	Day 2	Day 3	Day 4	Day 5	* p-value
	Median (min–max)				
Phonemic fluency	45 (40–48)	41 (39–45)	41 (40–45)	44 (41–48)	<0.001
Semantic fluency	25 (24–27)	22 (21–23)	22 (22–24)	26 (24–27)	<0.001
Forward DGS	5 (4–7)	4 (4–5)	4 (4–6)	6 (5–7)	<0.001
Backward DGS	5 (5–6)	4 (3–4)	4 (4–5)	6 (5–7)	<0.001
Logical memory subtest WMS-R	20 (20–22)	18 (18–19)	19 (18–20)	21 (20–22)	<0.001
VAS (dysmenorrhea)	6 (4–7)	8 (7–10)	7 (6–9)	4 (3–6)	<0.001

* Kruskal–Wallis post hoc test, DGS: digit span, VAS: visual analog scale.

**Table 2:** Comparison of the cognitive function scores on the most painful and least painful days.

Variables	Most painful day Day 3	Least painful day Day 5	* p-value
Phonemic fluency	41 (39–45)	44 (41–48)	<0.001
Semantic fluency	22 (21–23)	26 (24–27)	<0.001
Forward DGS	4 (4–5)	6 (5–7)	<0.001
Backward DGS	4 (3–4)	6 (5–7)	<0.001
Logical memory subtest WMS-R	18 (18–19)	21 (20–22)	<0.001

* Mann Whitney U test.

**Table 3:** Comparison of the phonemic fluency and semantic fluency scores across the days.

Variables	VAS day 2	VAS day 3	VAS day 4	VAS day 5
Phonemic fluency day 2	−0.9	-	-	-
Phonemic fluency day 3	-	−0.2	-	-
Phonemic fluency day 4	-	-	0.07	-
Phonemic fluency day 5	-	-	-	0.1
Semantic fluency day 2	−0.8	-	-	-
Semantic fluency day 3	-	−0.3	-	-
Semantic fluency day 4	-	-	0.06	-
Semantic fluency day 5	-	-	-	0.2

* Spearman correlation test.

**Table 4:** Comparison of the forward and backward DGS scores across the days.

Variables	VAS day 2	VAS day 3	VAS day 4	VAS day 5
Forward DGS day 2	−0.8	-	-	-
Forward DGS day 3	-	−0.1	-	-
Forward DGS day 4	-	-	0.07	-
Forward DGS day 5	-	-	-	−0.04
Backward DGS day 2	−0.5	-	-	-
Backward DGS day 3	-	−0.2	-	-
Backward DGS day 4	-	-	0.09	-
Backward DGS day 5	-	-	-	−0.04

* Spearman correlation test.

**Table 5:** Logical memory subtest WMS-R scores across the days.

Variables	VAS day 2	VAS day 3	VAS day 4	VAS day 5
Logical memory subtest WMS-R day 2	−0.5	-	-	-
Logical memory subtest WMS-R day 3	-	−0.1	-	-
Logical memory subtest WMS-R day 4	-	-	−0.2	-
Logical memory subtest WMS-R day 5	-	-	-	0.03

* Spearman correlation test.

## References

[1] MacGregor B Allaire C Bedaiwy MA Yong PJ Bougie O Disease burden of dysmenorrhea: impact on life course potential International Journal of Women’s Health 2023 15 499 509 10.2147/IJWH.S380006 PMC10081671 37033122

[2] Ferries-Rowe E Corey E Archer JS Primary dysmenorrhea: diagnosis and therapy Obstetrics and Gynecology 2020 136 5 1047 1058 10.1097/AOG.0000000000004096 33030880

[3] Kang HJ Dysmenorrhea Chung PH Rosenwaks Z Problem-focused reproductive endocrinology and infertility Contemporary Endocrinology Cham, Switzerland Springer 2023 47 51 10.1007/978-3-031-19443-6_7

[4] Atlıhan U Yavuz O Avşar HA Ata C Erkılınç S Vitamin D evaluation in adenomyosis: a retrospective cross-sectional study Turkish Journal of Obstetrics and Gynecology 2024 21 2 98 103 10.4274/tjod.galenos.2024.41662 38853492 PMC11589221

[5] Atlıhan U Ertan B Özgözen E Güney M Comparison of patients with adenomyosis detected in hysterectomy material and patients with other benign pathologies: retrospective study Turkish Journal of Reproductive Medicine and Surgery 2024 8 2 54 62 10.24074/tjrms.2023-100472

[6] Karout S Soubra L Rahme D Karout L Khojah HMJ Prevalence, risk factors, and management practices of primary dysmenorrhea among young females BMC Women’s Health 2021 21 1 14 10.1186/s12905-021-01532-w 34749716 PMC8576974

[7] Fernández-Martínez E Onieva-Zafra MD Parra-Fernández ML The impact of dysmenorrhea on quality of life among spanish female university students International Journal of Environmental Research and Public Health 2019 16 5 713 10.3390/ijerph16050713 30818861 PMC6427338

[8] Shewte MK Sirpurkar MS Dysmenorrhea and quality of life among medical and nursing students: a cross-sectional study National Journal of Community Medicine 2016 7 06 474 479 https://njcmindia.com/index.php/file/article/view/980

[9] Unsal A Ayranci U Tozun M Arslan G Calik E Prevalence of dysmenorrhea and its effect on quality of life among a group of female university students Upsala Journal of Medical Sciences 2010 115 2 138 145 10.3109/03009730903457218 20074018 PMC2853792

[10] Mendiratta V Primary and secondary dysmenorrhea, premenstrual syndrome, and premenstrual dysphoric disorder Lobo RA Gershenson DM Lentz GM Comprehensive Gynecology 7th ed Philadelphia, USA Elsevier Inc 2017 815 828

[11] Bernardi M Lazzeri L Perelli F Reis FM Petraglia F Dysmenorrhea and related disorders F1000Res 2017 6 1645 10.12688/f1000research.11682.1 28944048 PMC5585876

[12] Burnett MA Antao V Black A Feldman K Grenville A Prevalence of primary dysmenorrhea in Canada Journal of Obstetrics and Gynaecology Canada 2005 27 8 765 770 10.1016/S1701-2163(16)30728-9 16287008

[13] Itani R Soubra L Karout S Rahme D Karout L Primary dysmenorrhea: pathophysiology, diagnosis and treatment updates Korean Journal of Family Medicine 2021 43 2 101 108 10.4082/kjfm.21.0103 PMC8943241 35320895

[14] Alldredge BK Corelli RL Guglelmo BJ Jacobson PA Kradjan WA Koda-Kimble and Young’s Applied Therapeutics: the Clinial Use of Drugs 10th ed Philadelphia Lippincott Williams and Wilkins 2013 1337 1357

[15] Pakpour AH Kazemi F Alimoradi Z Griffiths MD Depression, anxiety, stress, and dysmenorrhea: a protocol for a systematic review Systematic Reviews 2020 9 65 1 6 10.1186/s13643-020-01319-4 32216827 PMC7098110

[16] Bajalan Z Moafi F MoradiBaglooei M Alimoradi Z Mental health and primary dysmenorrhea: a systematic review Journal of Psychosomatic Obstetrics and Gynecology 2018 40 3 185 194 10.1080/0167482X.2018.1470619 29745745

[17] van der Leeuw G Eggermont LHP Shi L Milberg WP Gross AL Pain and cognitive function among older adults living in the community Journals of Gerontology Series A: Biomedical Sciences and Medical Sciences 2016 71 3 398 405 10.1093/gerona/glv166 PMC5013972 26433218

[18] Orla M McGuire BE Finn DP The effect of pain on cognitive function: a review of clinical and preclinical research Progress in Neurobiology 2011 93 3 385 404 10.1016/j.pneurobio.2011.01.002 21216272

[19] van der Leeuw G Ayers EG Leveille S Blankenstein AH van der Horst H The effect of pain on major cognitive impairment in older adults The Journal of Pain 2018 19 12 1435 1444 10.1016/j.jpain.2018.06.009 30004021

[20] Keklicek H Aydın NS Can HB Aydın DD Kayatekin AY Dysmenorrhea and multitasking ability Gait and Posture 2020 81 1 183 10.1016/j.gaitpost.2020.07.127 32758918

[21] Keogh E Cavill R Moore D Eccleston C The effects of menstrual-related pain on attentional interference Pain 2014 155 4 821 827 10.1016/j.pain.2014.01.021 24486337

[22] Munro AK Hunter EC Hossain SZ Keep M A systematic review of the menstrual experiences of university students and the impacts on their education: A global perspective PLoS One 2021 16 9 e0257333 10.1371/journal.pone.0257333 34506544 PMC8432759

[23] Osayande A Mehulic S Diagnosis and initial management of dysmenorrhea American Family Physician 2014 89 5 341 346 24695505

[24] Lezak MD Executive functions and motor performance Neuropsychological Assessment 1995 650 685

[25] Wechsler D WMS-R: Wechsler memory scale-revised Psychological Corporation 1987

[26] Karakaş S Kafadar H Eski R Wechsler bellek ölçeği geliştirilmiş formunun test-tekrar test güvenirliği Türk Psikoloji Dergisi 1996 11 38 46 52 (in Turkish)

[27] Wu M Liang Y Wang Q Zhao Y Zhou R Emotion dysregulation of women with premenstrual syndrome Scientific Reports 2016 6 6 38501 10.1038/srep38501 27922107 PMC5138621

[28] Alanoğlu E Ulaş UH Özdağ F Odabaşı Z Cakçı A Auditory event-related brain potentials in fibromyalgia syndrome Rheumatology International 2005 25 5 345 349 10.1007/s00296-004-0443-3 14986061

[29] Maki PM Rich JB Rosenbaum RS Implicit memory varies across the menstrual cycle: estrogen effects in young women Neuropsychologia 2002 40 518 529 10.1016/S0028-3932(01)00126-9 11749982

[30] Bosma FK Kessels RP Cognitive impairments, psychological dysfunction, and coping styles in patients with chronic whiplash syndrome Neuropsychiatry, Neuropsychology and Behavioral Neurology 2002 15 1 56 65 11877552

[31] Bäckman L Dixon RA Psychological compensation: a theoretical framework Psychological Bulletin 1992 112 2 259 283 10.1037//0033-2909.112.2.259 1454895

[32] Barnard K Frayne S Skinner K Sullivan L Health status among women with menstrual symptoms Journal of Women’s Health 2003 12 9 911 919 10.1089/154099903770948140 14670171

[33] Bair MJ Robinson RL Katon W Kroenke K Depression and pain comorbidity: a literature review Archives of Internal Medicine 2003 163 20 2433 2445 10.1001/archinte.163.20.2433 14609780

[34] Zis P Daskalaki A Bountouni I Sykioti P Varrassi G Depression and chronic pain in the elderly: links and management challenges Clinical Interventions in Aging 2017 12 709 720 10.2147/cia.s113576 28461745 PMC5407450

[35] Gagua T Tkeshelashvili B Gagua D Mchedlishvili N Assessment of anxiety and depression in adolescents with primary dysmenorrhea: a case-control study Journal of Pediatric and Adolescent Gynecology 2013 26 6 350 354 10.1016/j.jpag.2013.06.018 24075089

[36] Lord T Taylor K Monthly fluctuation in task concentration in female college students Perceptual and Motor Skills 1991 72 2 435 439 10.2466/pms.1991.72.2.435 1852554

[37] Hampson E Kimura D Reciprocal effects of hormonal fluctuations on human motor and perceptual-spatial skills Behavioral Neuroscience 1988 102 3 456 459 10.1037/0735-7044.102.3.456 3395456

